# Supplementary material for: Effects of COVID-19 vaccination and previous infection on Omicron SARS-CoV-2 infection and relation with serology
Source: Nat Commun. 2023 Aug 9;14:4793. doi: 10.1038/s41467-023-40195-z (PMC10412579; doi:10.1038/s41467-023-40195-z)
Supplement: Supplementary file 1 — Supplementary Information [file 41467_2023_40195_MOESM1_ESM.pdf]

Supplementary material

To manuscript

Effects of COVID-19 vaccination and previous infection on Omicron SARS-CoV-2 infection and relation with serology

Brechje de Gier, Anne J. Huiberts, Christina E. Hoeve, Gerco den Hartog, Henri van Werkhoven, Rob van Binnendijk, Susan J.M. Hahné, Hester E. de Melker, Susan van den Hof, Mirjam J. Knol

Table S1. Vaccines received by study participants, per dose. Participants who did not receive the dose were excluded from the percentage calculation.

		**N (%)**
**Dose 1**	AstraZeneca (Vaxzevria)	14585 (34.5)
	BioNTech/ Pfizer (Comirnaty)	17036 (40.3)
	Janssen	4729 (11.2)
	Moderna (Spikevax)	5864 (13.9)
	Unknown	31 (0.1)
**Dose 2**	AstraZeneca (Vaxzevria)	13505 (32.3)
	BioNTech/ Pfizer (Comirnaty)	19191 (45.9)
	Janssen	115 (0.3)
	Moderna (Spikevax)	7971 (19.0)
	Novavax	2 (0.0)
	Unknown	1072 (2.6)
**Dose 3**	AstraZeneca (Vaxzevria)	343 (1.0)
	BioNTech/ Pfizer (Comirnaty)	13822 (38.5)
	Janssen	18 (0.1)
	Moderna (Spikevax)	21408 (59.7)
	Novavax	2 (0.0)
	Unknown	264 (0.7)
**Dose 4**	AstraZeneca (Vaxzevria)	99 (0.8)
	BioNTech/ Pfizer (Comirnaty)	3239 (27.3)
	Janssen	4 (0.0)
	Moderna (Spikevax)	8159 (68.7)
	Novavax	5 (0.0)
	Unknown	374 (3.1)

Table S2. Association between anti-S-antibody concentration in quartiles and risk of infection, crude and adjusted for age, sex, educational level and medical risk group, n = 20,402 participants.

**Quartile log-anti-S-antibody concentration**	**Person-days**	**Infections**	**HR (95% CI) crude**	**HR (95% CI) adjusted**
**(3,8.82]**	101221	318	[ref]	[ref]
**(8.82,9.43]**	102668	251	0.75 (0.64-0.89)	0.83 (0.70-0.98)
**(9.43,10.4]**	101896	203	0.59 (0.49-0.70)	0.65 (0.55-0.79)
**(10.4,12.3]**	102326	78	0.24 (0.19-0.31)	0.29 (0.23-0.37)

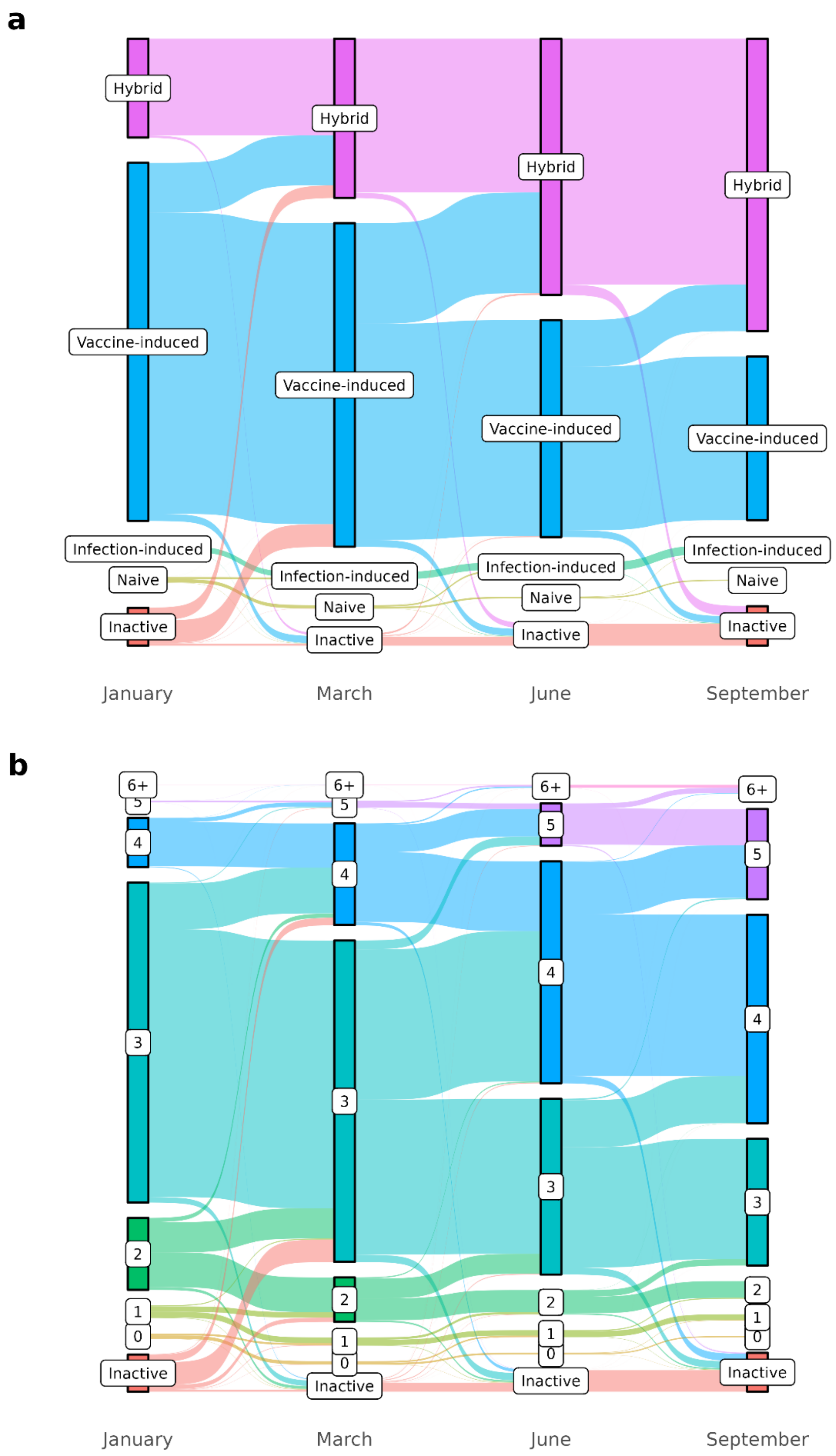

Figure S1. Sankey diagrams showing the changes in the VASCO study population during the study period (January-September 2022), in terms of type of immunity (A) and number of prior immunizing events (B).

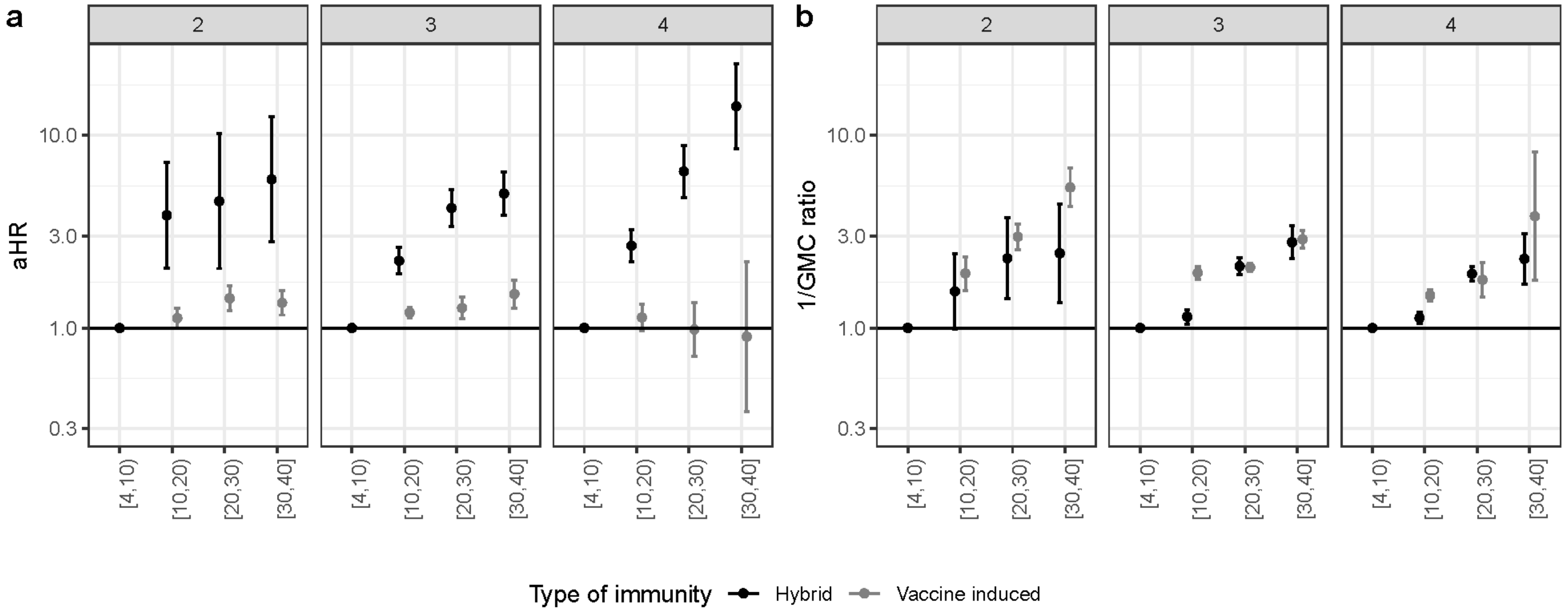

Figure S2. A. Adjusted hazard ratio (aHR) of infection with Omicron SARS-CoV-2 by type of immunizing events, stratified by the number of immunizing events, n = 39,810 participants. B. One divided by the adjusted geometric mean concentration (GMC) ratio of S-antibodies, n = 20,402 participants. In both analyses, 4 to 10 weeks after the last immunizing event was the reference group. The group with 4 immunizing events only includes participants aged 60 and older, because younger people were not eligible for 4 vaccinations. Data are presented as aHR with 95% confidence intervals, adjusted for age, sex, educational level and medical risk group. Numbers between brackets on the x axis represent weeks since the last immunizing event.

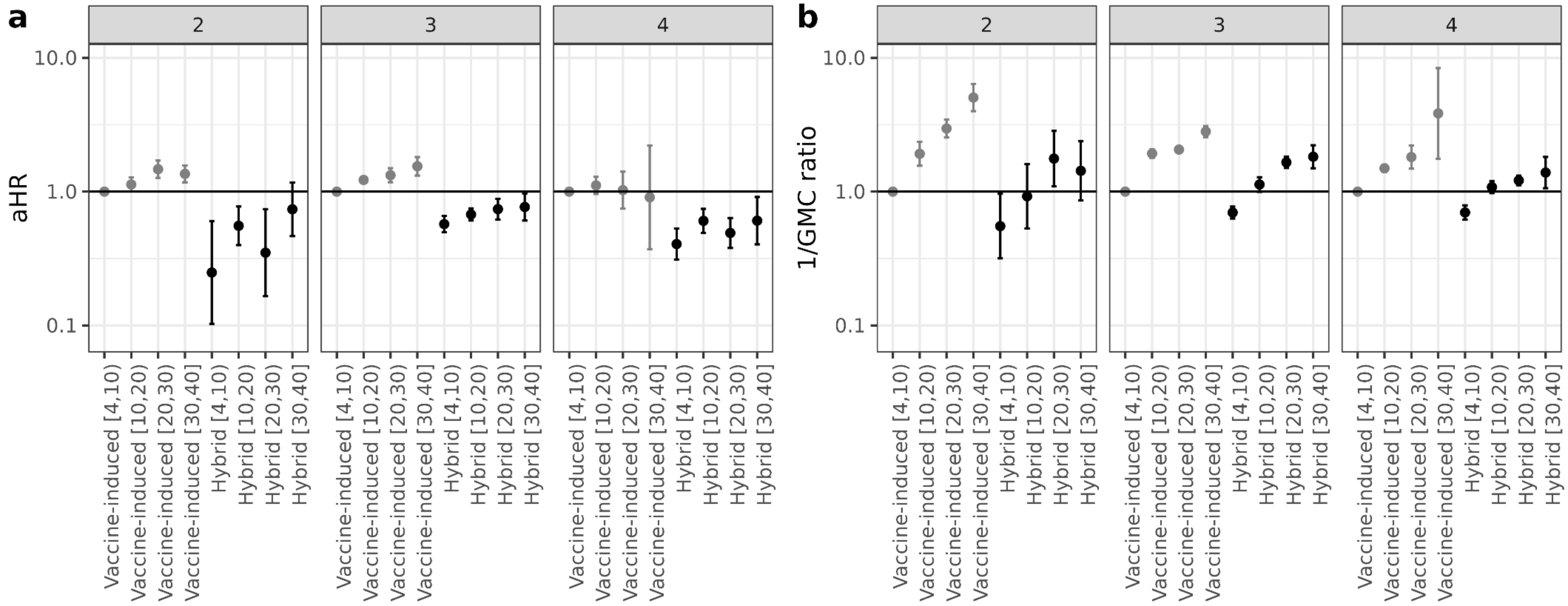

Figure S3: A. Adjusted hazard ratio (HR) of infection with Omicron SARS-CoV-2 by type of immunizing event, stratified by the number of immunizing events, excluding participants with a previous Omicron infection, n = 38,155 participants. B. One divided by the adjusted geometric mean concentration (GMC) ratio of S-antibodies, excluding participants with a previous Omicron infection, n = 15,811 participants. In both analyses, 4 to 10 weeks after the last vaccination for vaccine-only immunity was the reference group. The group with 4 immunizing events only includes participants aged 60 and older, because younger people were not eligible for 4 vaccinations. Data are presented as aHR with 95% confidence intervals, adjusted for age, sex, educational level and medical risk group. Numbers between brackets on the x axis represent weeks since the last immunizing event.
