# Supplementary material for: Effects of COVID-19 vaccination and previous infection on Omicron SARS-CoV-2 infection and relation with serology
Source: Nat Commun. 2023 Aug 9;14:4793. doi: 10.1038/s41467-023-40195-z (PMC10412579; doi:10.1038/s41467-023-40195-z)
Supplement: Supplementary file 3 — Description of Additional Supplementary Files [file 41467_2023_40195_MOESM3_ESM.pdf]

Description of Additional Supplementary Files

File Name: Supplementary Data 1
Description: Crude and adjusted hazard ratios of infection with Omicron SARS-CoV-2 by hybrid or vaccine-induced immunity, and crude and adjusted geometric mean concentration (GMC) ratio of S-antibodies, stratified by the number of prior immunizing events. In both analyses, 4 to 10 weeks after the last vaccination for vaccine-only immunity was the reference group. The group with 4 immunizing events only includes participants aged 60 and older, because younger people were not eligible for 4 vaccinations. Data are presented with 95% confidence intervals and adjustment was done for age, sex, educational level and medical risk group

File Name: Supplementary Data 2
Description: Crude and adjusted hazard ratios of infection with Omicron SARS-CoV-2 by the type of first and last immunizing event, stratified by the number of exposures. 4 to 10 weeks after the last vaccination for infection first and vaccination last was the reference. Data are presented with 95% confidence intervals and adjustment was done for age, sex, educational level and medical risk group.

File Name: Supplementary Data 3
Description: Crude and adjusted hazard ratios of infection with Omicron SARS-CoV-2 by number of immunizing events. 4 to 10 weeks after the third immunizing event was the reference group. Data are presented with 95% confidence intervals and adjustment was done for age, sex, educational level and medical risk group.
