# Supplementary material for: Effects of COVID-19 vaccination and previous infection on Omicron SARS-CoV-2 infection and relation with serology
Source: Nat Commun. 2023 Aug 9;14:4793. doi: 10.1038/s41467-023-40195-z (PMC10412579; doi:10.1038/s41467-023-40195-z)
Supplement: Supplementary file 7 — Reporting Summary [file 41467_2023_40195_MOESM7_ESM.pdf]

Corresponding author(s): Mirjam Knol

Last updated by author(s): Jul 12, 2023

Reporting Summary

Nature Portfolio wishes to improve the reproducibility of the work that we publish. This form provides structure for consistency and transparency in reporting. For further information on Nature Portfolio policies, see our Editorial Policies and the Editorial Policy Checklist.

Statistics

For all statistical analyses, confirm that the following items are present in the figure legend, table legend, main text, or Methods section.

n/a	Confirmed	
☐	☒	The exact sample size (*n*) for each experimental group/condition, given as a discrete number and unit of measurement
☐	☒	A statement on whether measurements were taken from distinct samples or whether the same sample was measured repeatedly
☒	☐	The statistical test(s) used AND whether they are one- or two-sided<br>*Only common tests should be described solely by name; describe more complex techniques in the Methods section.*
☐	☒	A description of all covariates tested
☐	☒	A description of any assumptions or corrections, such as tests of normality and adjustment for multiple comparisons
☐	☒	A full description of the statistical parameters including central tendency (e.g. means) or other basic estimates (e.g. regression coefficient) AND variation (e.g. standard deviation) or associated estimates of uncertainty (e.g. confidence intervals)
☒	☐	For null hypothesis testing, the test statistic (e.g. *F*, *t*, *r*) with confidence intervals, effect sizes, degrees of freedom and *P* value noted<br>*Give P values as exact values whenever suitable.*
☒	☐	For Bayesian analysis, information on the choice of priors and Markov chain Monte Carlo settings
☒	☐	For hierarchical and complex designs, identification of the appropriate level for tests and full reporting of outcomes
☒	☐	Estimates of effect sizes (e.g. Cohen's *d*, Pearson's *r*), indicating how they were calculated

Our web collection on statistics for biologists contains articles on many of the points above.

Software and code

Policy information about availability of computer code

Data collection	Questionnaire data is collected through a (mobile) app of YourResearch (www.yourresearch.com).
Data analysis	Analyses were done with R version 4.3.0, using packages Epi (version 2.47.1), survival (version 3.5-5) and stats (version 4.3.0).

For manuscripts utilizing custom algorithms or software that are central to the research but not yet described in published literature, software must be made available to editors and reviewers. We strongly encourage code deposition in a community repository (e.g. GitHub). See the Nature Portfolio guidelines for submitting code & software for further information.

Data

Policy information about availability of data

All manuscripts must include a data availability statement. This statement should provide the following information, where applicable:

- Accession codes, unique identifiers, or web links for publicly available datasets
- A description of any restrictions on data availability
- For clinical datasets or third party data, please ensure that the statement adheres to our policy

The data underlying Figures 1-3 generated in this study have been deposited in the Data S1, S2 and S3 files. The anonymized data reported in this study can be obtained from the corresponding author upon request. The dataset may include individual data and a data dictionary will be provided. Data requests should include a proposal for the planned analyses. Decisions will be made according to data use by the statistical disclosure working group within RIVM. Data transfer will require a signed data sharing agreement.

Research involving human participants, their data, or biological material

Policy information about studies with human participants or human data. See also policy information about sex, gender (identity/presentation), and sexual orientation and race, ethnicity and racism.

Reporting on sex and gender	Participants self-reported their sex, with categories Male, Female and Other. This variable was included as covariate in all models. Descriptive results are reported in Table 1.
Reporting on race, ethnicity, or other socially relevant groupings	Highest attained level of education, as self-reported by the participant, was included as covariate in all models.
Population characteristics	Table 1 of the manuscript provides descriptive statistics on the participants (agegroup, sex, education level and medical risk group).
Recruitment	VASCO participants were recruited through a random draw from the population register and through (social) media campaigns and inclusion ran from May 2021 and December 2021. Older persons were oversampled in VASCO. Also, due to self-selection to participate in the study, VASCO participants are more often women and are more often highly educated compared to the overall population in the Netherlands. Participants were also more often of Dutch ethnicity. Oversampling of older people and self-selection might have affected the generalizability of our results to the Dutch population.
Ethics oversight	The VASCO study was approved by the Medical Ethics Committee of the Stichting Beoordeling Ethiek Biomedisch Onderzoek (BEBO), Assen, the Netherlands (NL76815.056.21).

Note that full information on the approval of the study protocol must also be provided in the manuscript.

Field-specific reporting

Please select the one below that is the best fit for your research. If you are not sure, read the appropriate sections before making your selection.

☒ Life sciences ☐ Behavioural & social sciences ☐ Ecological, evolutionary & environmental sciences

For a reference copy of the document with all sections, see nature.com/documents/nr-reporting-summary-flat.pdf

Life sciences study design

All studies must disclose on these points even when the disclosure is negative.

Sample size	During the study period, 43,257 participants contributed 8,291,966 person-days. As the cohort was not specifically designed for this substudy, this pertains a convenience sample.
Data exclusions	Participants were excluded if they reported to have received more doses than possible according to the Dutch vaccination strategy. No further data exclusions apply.
Replication	Within our study group, researchers have successfully replicated the analyses.
Randomization	Not applicable, as the study is observational.
Blinding	Not relevant, analysis of data gathered in an ongoing observational study.

Reporting for specific materials, systems and methods

We require information from authors about some types of materials, experimental systems and methods used in many studies. Here, indicate whether each material, system or method listed is relevant to your study. If you are not sure if a list item applies to your research, read the appropriate section before selecting a response.

Materials & experimental systems

n/a	Involved in the study	
☐	☒	Antibodies
☒	☐	Eukaryotic cell lines
☒	☐	Palaeontology and archaeology
☒	☐	Animals and other organisms
☐	☒	Clinical data
☒	☐	Dual use research of concern
☒	☐	Plants

Methods

n/a	Involved in the study	
☒	☐	ChIP-seq
☒	☐	Flow cytometry
☒	☐	MRI-based neuroimaging

Antibodies

Antibodies used	Serum samples were analyzed with the Elecsys Anti-SARS-CoV-2 S and Anti-SARS-CoV-2 assays on the Cobas e801 (Roche Diagnostics, Mannheim, Germany), which are electrochemiluminescence immunoassays measuring Ig levels against respectively the receptor binding domain of the Spike (S1) protein (S-antibodies) and the Nucleoprotein (N-antibodies) of SARS-COV-2.
Validation	Sensitivity and specificity of the used assays are described on the manufacturer's website: https://diagnostics.roche.com/global/en/products/params/elecsys-anti-sars-cov-2.html and https://diagnostics.roche.com/global/en/products/params/elecsys-anti-sars-cov-2-s.html.

Clinical data

Policy information about clinical studies

All manuscripts should comply with the ICMJE guidelines for publication of clinical research and a completed CONSORT checklist must be included with all submissions.

Clinical trial registration	Dutch Trial Register (NTR), registration number NL9279 (available via ICTRP Search Portal (who.int))
Study protocol	https://www.onderzoekmetmensen.nl/en/trial/26317
Data collection	Inclusion from May 2021 to December 2022. Data collection is ongoing. Data is collected by the participant at home.
Outcomes	The primary endpoint is time to first symptomatic SARS-CoV-2 infection, determined by a positive PCR or antigen test in combination with COVID-19 related symptoms. Data on the primary outcome were collected through questionnaires filled out by the participants.<br><br>Secondary endpoints that are investigated:<br>- SARS-CoV-2 infections by disease severity: COVID-19 related death, COVID-19 related hospitalization, mild SARS-CoV-2 infection, asymptomatic SARS-CoV-2 infection<br>- Unsolicited adverse events for which medical attention was sought<br>Data on the secondary outcomes were also collected through questionnaires filled out by the participants. COVID-19 related death has not been ascertained yet but will be assessed through linkage with the 'cause-of-death' register, for participants who gave informed consent for linkage at the start of the study.
